# Prevalence of malaria in Woreta town, Amhara region, Northwest Ethiopia over eight years

**DOI:** 10.1186/s12889-018-5913-8

**Published:** 2018-08-08

**Authors:** Amir Alelign, Zinaye Tekeste, Beyene Petros

**Affiliations:** 1 Department of Microbial, Cellular and Molecular Biology, Addis Ababa University, P.O.Box 1176, Addis Ababa, Ethiopia; 2 Department of Biology, College of Computational and Natural Sciences, Debrebirhan University, Debrebirhan, Ethiopia; 3 Aklilu Lemma Institute of Pathobiology, Addis Ababa University, P.O. Box 1176, Addis Ababa, Ethiopia

**Keywords:** Malaria, Prevalence, Retrospective, Woreta, Ethiopia

## Abstract

**Background:**

Data on trends in malaria prevalence is significant to assist efforts in the control and prevention of the disease. This retrospective study was, therefore, aimed to determine the prevalence of malaria in Woreta town, northwestern Ethiopia over 8 years.

**Methods:**

A retrospective study was conducted in Woreta town, northwestern Ethiopia, from November to January 2013. Eight years (2005 to 2012) health center record of malaria cases was reviewed. Odds ratio (OR) was used to determine trend in malaria prevalence with respect to age, sex and *Plasmodium* species. *P*-values less than 0.05 were considered to be statistically significant.

**Results:**

From 2005 to 2012, a total of 102,520 suspected cases of malaria were reported at Woreta health center. Of these, 33,431 (32.6%) were microscopically confirmed to be positive for the disease. Among these positive cases, 17,700 (52.9%) and 15,731 (47.1%) were males and females, respectively. Children less than 5 years old were 1.3 times more likely to be infected by malaria than those with 5–15 years ([OR]; 1.3, 95% confidence interval [CI]; 1.26–1.34, *p* &lt; 0.001). There was higher percentage (69.7%) of *Plasmodium falciparum* infection than *Plasmodium vivax* (26.5%); and the difference was statistically significant (*p* &lt; 0.05). There was fluctuation in yearly malaria prevalence with a minimum of 7% in 2008 and maximum of 47% in 2005.

**Conclusions:**

The present study revealed that malaria continued to be one of the major public health problems in Woreta town, northwest Ethiopia. Moreover, there was no successive yearly reduction in its prevalence. Therefore, efforts are required to reduce the disease burden through continuous monitoring and evaluation of control measures in the study area.

## Background

Global malaria prevention and control have been scaled up in the past decade, with notable progress in sub-Saharan Africa. [1]. However, the transmission of the diseases remained active in many countries around the world. By the end of 2015, there were 95 countries and territories with ongoing malaria transmission. Globally, an estimated 214 million cases and 438,000 deaths occurred due to malaria in the same year. Most cases and deaths were estimated to have occurred in the African region (88%), followed by the South-East Asia region [2].

In Ethiopia, malaria remained to be the leading communicable disease seen at health facilities [3]. It accounted for up to 14% of outpatient consultations and 9% of health facility admissions. It has been estimated that there were 5–10 million clinical malaria cases and approximately 70,000 people die of it each year in the country [4].

Malaria has continued to be one of the major public health challenges in the Amhara region, where children and pregnant women were severely affected [5]. A total of 1,127,241 cases of malaria were reported within the region in 2012. Out of eight zones in the region, only five of them accounted for 93.1% of the total malaria burden. South Gondar zone accounted for the third greatest number of cases in the region next to West Gojam and North Gondar zones. Malaria prevalence declined significantly in the Amhara region from 4.6% in 2006 to 0.6% in 2007 and 0.8% in 2011 [6].

In the Amhara region, prevention and control activities of the disease have been implemented as guided by the National Strategic Plan: A combination intervention strategy including early diagnosis and prompt treatment, selective vector control that involved use of indoor residual spraying (IRS), insecticide-treated mosquito nets (ITNs) and environmental management [7]. However, malaria control in the country as a whole and in the region particularly continued to experience many problems.

Studies have shown that the number of malaria cases and plasmodium species composition vary over time. This variation was suggested to be due to climatic, intervention measures, environmental or human behavioral risk factors in those years [8–12].

Assessment of the pattern of malaria burden in the past years and comparing it to the present will help to evaluate the effectiveness of proven control interventions of the disease in a locality [12]. However, much work has not been done to this extent in malaria endemic regions of Ethiopia such as Woreta. Therefore, the present study aimed to assess the prevalence of malaria in person and *Plasmodium* species composition in the past 8 years (2005–2012) in Woreta.

## Methods

### Study area

The study was conducted at Woreta town, Fogera district, Amhara region, northwest Ethiopia. The area is located 625 km north of Addis Ababa (the capital of Ethiopia). The district has been divided in to 30 rural and 5 urban ‘*kebeles’* (the smallest administrative unit) in the district and Woreta and Alember were the major towns. The total human population of the district was 233,529, of which, 26,812 was urban population. Fogera has been known for its flat and low land. The district has been regarded as malarious. The altitude of the district ranged between 1750 and 2100 m above sea level. The capital of the district, Woreta town, had an altitude and longitude of 11^o^55’N 37^o^42’E with an elevation of 1828 m above sea level. Mean annual precipitation was 1225.8 mm with mean maximum and minimum temperatures of 27.9 and 12.6 °C. The major portion of the total annual rain fall was received between June and October [13]. Malaria has been the most prevalent seasonal disease in the area, and October to December was the peak transmission season.

### Study design and population

A retrospective study was conducted to determine prevalence of malaria in Woreta town over 8 years (2005–2012). The town has been divided in to four ‘kebeles’. The study population included residents of the four ‘Kebeles’ in the town who had visited the health center during the study period and suspected of malaria.

### Data collection

An 8 years (2005–2012) retrospective data on malaria prevalence was collected from May to July 2013 at Woreta health center. In this health center, peripheral smear examination of blood film has been used as the gold standard in confirming the presence of malaria infection.

### Data quality control

The retrospective data were taken from the primary record book of the health center. The information of every member of the community was registered during their visit to the health center within the study period. Three trained health workers collected the data independently and cross cheeked each other and finally confirmed by the first author.

### Data analysis

The data were entered in Microsoft Excel data sheets, cross checked and transferred, and analyzed using SPSS 16 software package. Odds ratio (OR) with the corresponding 95% confidence interval (CI) was used to determine the differences in malaria prevalence and *Plasmodium* species distribution among the different age groups and sexes over time. *P*-values &lt; 0.05 were considered to be statistically significant.

## Results

A total of 102,520 blood films were examined for malaria from 2005 to 2012 at Woreta health center. Of these, 33,431 [17,700 (52.9%) males and 15,731 (47.1%) females] were positive for malaria (Table 1). About 52.6, 23.5 and 23.9% of individuals in the &gt; 15, &lt; 5 and from 5 to 15 years age groups were malaria infected, respectively. Totally, malaria infection in children &lt; 5 years was significantly lower than that of individuals with 5 to 15 and &gt; 15 years age groups[(&lt; 5 years vs. 5–15 years, OR: 1.30, CI: 1.26–1.34, *p* &lt; 0.05) ((&lt; 5 years vs. &gt; 15 years, OR: 1.29, CI: 1.24–1.33, *p* &lt; 0.05)] (Table 1).

**Table 1:** Distribution of malaria cases by sex and age, Woreta town, Northwest Ethiopia. (2005–2012)

Malaria cases					
Sex	Age groups (years)	Positive (%)	Negative(%)	OR (95% CI)	*p*-value
Males	&lt; 5	3304 (18.7)	8075 (23.4)	1.00	
	5–15	10,027 (56.6)	17,995 (52.1)	1.36 (1.30–1.40)	&lt; 0.001
	&gt; 15	4369 (24.7)	8449 (24.5)	1.26 (1.20–1.34)	&lt; 0.001
	Sub- total	17,700 (100.0)	34,519 (100.0)		
Females	&lt; 5	4558 (29.0)	11,658 (33.9)	1.00	
	5–15	7565 (48.1)	15,656 (45.5)	1.24 (1.18–1.30)	&lt; 0.001
	&gt; 15	3608 (22.9)	7085 (20.6)	1.30 (1.24–1.40)	&lt; 0.001
	Sub- total	15,731 (100.0)	34,401 (100.0)		
Total	&lt; 5	7862 (23.5)	19,639 (28.4)	1.00	
	5–15	17,592 (52.6)	33,912 (49.1)	1.30 (1.26–1.34)	&lt; 0.001
	&gt; 15	7977 (23.9)	15,538 (22.5)	1.29 (1.24–1.33)	&lt; 0.001
	Total	33,431 (100.0)	69,089 (100.0)		

*CI* confidence interval, *OR* odds ratio; &lt; 5 and &lt; 15 indicated study subjects who were less than 5 and 15 years of ages in both sexes, respectively; &gt; 15 indicated those study subjects who were above 15 years of ages

Of the total malaria cases identified, there was significantly higher *P.falciparum* (69.7%) infection than *P.vivax* (26.5%) (*p* &lt; 0.05). Mixed infection accounted only for 3.8% of the total cases. Higher percentage of *P.falciparum* infection was recorded in all age groups and in both sexes. Totally, infections by *P. falciparum* and *P.vivax* malaria were about 2.7 (OR: 2.66; CI: 2.46–2.89; *p* &lt; 0.001) and 3.0 (OR: 2.99; CI: 2.76–3.26; *p* &lt; 0.001) times more likely to occur than mixed (both *P. falciparum* and *P. vivax*) infections, respectively (Table 2).

**Table 2:** Distribution of malaria cases by age groups and species, Woreta town, northwest Ethiopia (2005–2012)

Malaria cases					
Age groups	Diagnosis	Positive (%)	Negative (%)	OR (95% CI)	*p*-value
&lt; 5	*P.falciparum*	5097 (64.8)	18,901(71.4)	7.04 (2.46–2.89)	&lt; 0.001
	*P.vivax*	2501 (31.8)	7432 (28.1)	5.64 (4.57–6.96)	&lt; 0.001
	Mixed	264 (3.4)	139 (0.5)	1.00	
	Sub-total	7862 (100.0)	26,472 (100.0)		
5–15	*P.falciparum*	12,194 (69.3)	26,065 (67.7)	2.32 (2.08–2.59)	&lt; 0.001
	*P.vivax*	4694 (26.7)	11,788 (30.6)	2.73 (2.44–3.05)	&lt; 0.001
	Mixed	704 (4.0)	647 (1.7)	1.00	
	Sub- total	17,592 (100.0)	38,500 (100.0)		
&gt; 15	*P.falciparum*	5983 (75.0)	12,057 (68.1)	1.61 (1.39–1.87)	&lt; 0.001
	*P.vivax*	1675 (21.9)	5260 (29.7)	2.51 (2.15–2.94)	&lt; 0.001
	Mixed	319 (3.1)	398 (2.2)	1.00	
	Sub- total	7977 (100.0)	17,715 (100.0)		
Total	*P.falciparum*	23,274 (69.7)	57,023 (69.0)	2.66 (2.46–2.89)	&lt; 0.001
	*P.vivax*	8870 (26.5)	24,480 (29.6)	2.99 (2.76–3.26)	&lt; 0.001
	Mixed	1287 (3.8)	1184 (1.4)	1.00	

*CI* confidence interval, *OR* Odds ratio; &lt; 5 and &lt; 15 indicated study subjects who were less than 5 and 15 years of ages in both sexes, respectively, &gt; 15 indicated those study subjects who were above 15 years of ages

**Fig. 1:**
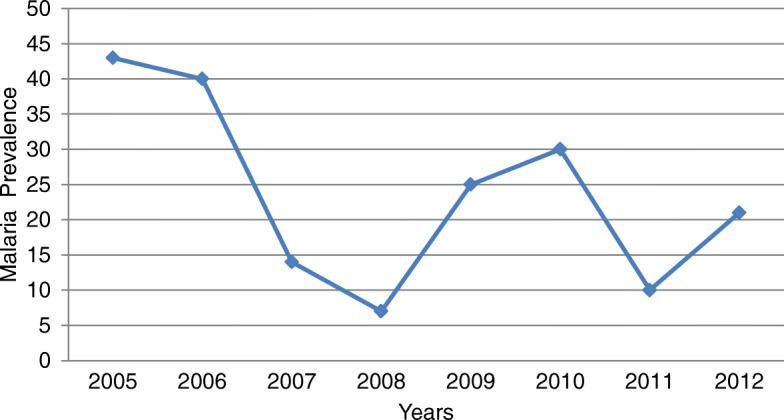
Annual trend of malaria prevalence at Woreta town, Northwest Ethiopia (2005–2012)

A fluctuating trend was observed in the yearly malaria prevalence. It was observed to range from 7% in 2008 to 43% in 2005. There were peaks in the prevalence in 2005 and 2010 (Fig. 1). There was a sharp increase of malaria prevalence from 2008 to 2010 and a fall from 2010 to 2011.

**Fig. 2:**
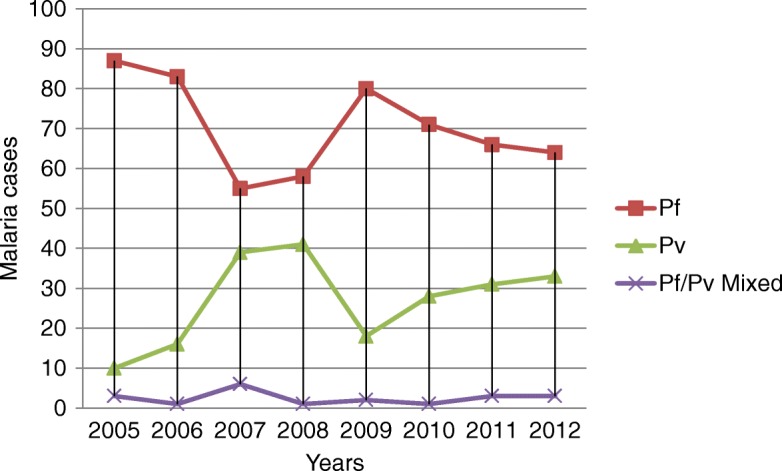
Trend of malaria cases by year and malaria species at Woreta town, Northwest Ethiopia (2005–2012). Pf*: Plasmodium falciparum,* Pv*: Plasmodium vivax,* Pf/pv mixed*:* co- infection of *Plasmodium falciparum and Plasmodium vivax*

Of the total malaria positive cases in 2005, about 77 (87%) and 9 (10%) were due to *P. falciparum* and *P. vivax,* respectively (Fig. 2). From 2009 to 2012, the prevalence of *P. falciparum* showed a decreasing pattern but that of *P. vivax* reflected a slight increment (18% in 2009, 28% in 2010, 31% in 2011 and 33% in 2012). The highest prevalence of mixed infection (6%) was observed in 2007 (Fig. 2).

**Fig. 3:**
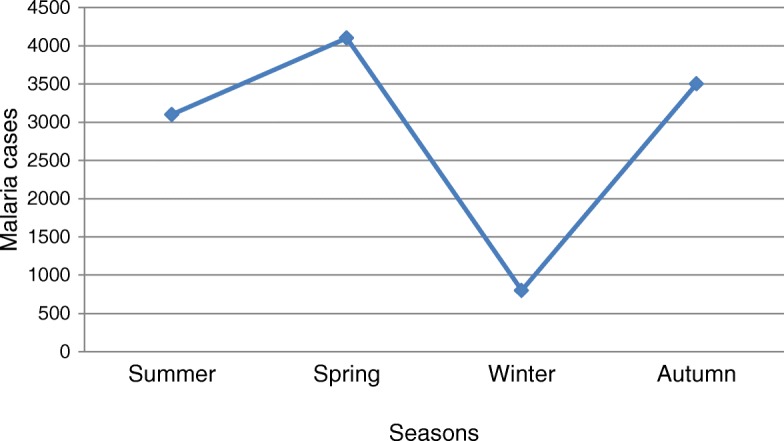
An eight years (2005–2012) average seasonal distribution of malaria cases at Woreta town, Northwest Ethiopia

There were two peaks of malaria season in the study area; one was in Autumn (March to May), and the other was in Spring (September to November). The highest (4100) and lowest (800) average cases of malaria were recorded during Spring and Winter (December to February), respectively (Fig. 3).

## Discussion

This study revealed individuals above 5 years of age were more affected than children below 5 years of age. This was in agreement with studies conducted in other parts of Ethiopia and South Africa [14, 15]. In contrast, higher prevalence of malaria was reported in children less than 5 years of age elsewhere in the country [16, 17]. The observed lower prevalence of malaria in children under 5 years of age might be because of their less likely exposure to infected mosquito bite due to good awareness and practices of their parents/care takers on malaria control and prevention activities. In addition, the partially acquired immunity developed during childhood in such high malaria transmission area might have a protective role in this age group. In high malaria transmission settings, partial immunity to the disease is acquired during childhood. In such areas, the majority of malarial disease, and particularly severe disease with rapid progression to death, occurs in young children without acquired immunity [18].

The finding that males were more affected than females in this study was in agreement with that of other studies conducted in different parts of Ethiopia [5, 10, 11]. In contrast, lower malaria cases in males were reported from studies in Kenya [19] and Mozambique [20]. This might be explained by the fact that in the present study area males were often engaged in early night outdoor agricultural activities, hence, have got higher chance of exposure to be infected by the anopheles malaria vector. Evidences showed that much greater mosquito human–biting activities occurred outdoors than indoors and during early parts of the night, suggesting higher outdoor malaria transmission potential in Ethiopia [21].

*P. falciparum* malaria was the major cause of morbidity in the region. This was in agreement with a similar study conducted in Northwestern Ethiopia which reported that *P. falciparum* and *P. vivax* accounted for 75 and 25% of malaria morbidity, respectively [7]. The observed higher prevalence of *falciparum* malaria in the study area was consistent with the Ethiopian Federal Ministry of Health (FMoH) report [22]. However, higher prevalence of *P. vivax* malaria was reported in Southern Ethiopia [11–13]. Although it was difficult to reason out the above differences, it might be due to differences in topographic adaptations and intrinsic factors of the parasites.

In the present study, a proportional raise in the prevalence of *P. vivax* was recorded from the year 2009 to 2012. An increased rate of drug resistance has been reported in the study region. Therefore, chloroquine resistant *vivax* malaria may be responsible for the observed slight increment in the proportion of *P. vivax* in the last few years. This was in agreement with a similar study conducted in Southern Ethiopia [10]. In agreement with the present study, high peaks of malaria incidence were reported in the same years in a similar study conducted in Southwestern part of the country [11].

Despite the fluctuating trend, a general reduction of the high malaria prevalence, 47% in 2005, 30% in 2010 and 21% in 2012 was recorded. This might be due to the continuous efforts made as part of the national malaria control program, which begun more than half a century ago [23]. This was in agreement with a study reported that since 2004, Ethiopia’s health systems for case management and surveillance have been greatly strengthened [24]. In particular, in the Amhara region, an integrated malaria vector control measures were made based on the national malaria control strategies. The national malaria control strategies were guided by the Abuja (Nigeria) declaration which targeted to reduce the burden of malaria in at risk groups such as children and pregnant women for the year 2005 [25]. This general reduction of malaria prevalence coupled with the launching of the extensive malaria control strategies in 2005 [4] demonstrated the effectiveness of the control measures implemented during those specified periods (2005–2010) in the study area.

However, despite all these efforts, malaria persisted as one of the major health problems in Woreta town. A 28% average malaria cases for the years 2005–2012 and a 21% in 2012 was recorded, which was far higher than the regional (4.6%) and the zonal (6.1%) malaria prevalence [26]. This was in parallel with the report from the Ethiopian Malaria Indicator Survey [21] and a similar study conducted in the same study area (Tsegaye T: Assessment of the efficacy of arthemeter-lumefantrine (Coartem®) for uncomplicated falciparum malaria at Woreta town, North Western Ethiopia, unpublished). In consistent to the findings of this study, similar works in other parts of East Africa revealed high prevalence of malaria in their respective study sites, 28% in Kenya and 38.1% in Tanzania [27, 28]. In contrary, a study conducted in southern Ethiopia reported a lower prevalence (0.93%) of malaria [8]. The above difference in malaria prevalence might be explained by the fact that malaria distribution varied at varying geographical locations and altitudes.

## Conclusions

A high prevalence of malaria was observed during the study period in Woreta town. A general reduction in malaria prevalence in the earlier years might have demonstrated the effectiveness of the malaria control program implemented in the area. However, in spite of integrated efforts made to control malaria and its vector, the disease persisted for more than half a century in the study area. Hence, more attention should be given in the practice and proper implementation of the malaria control activities in the local community. Further studies are needed to evaluate the independent effect of other possible special factors such as the ongoing massive rice irrigation activities in the study area, which might favor the reproduction of malaria mosquito vectors.

### Limitation

The present study was restricted to the health center recorded retrospective data and was unable to relate to the current prevalence status of malaria in the study area.

## Abbreviations

CI: Confidence interval
FMoH: Federal ministry of health
IRS: Indoor residual spraying
ITNs: Insecticide-treated mosquito nets
OR: Odds ratio
SPSS: Statistical package for the social sciences
